# *Notice to Readers*: Change in *MMWR* Announcements

**DOI:** 10.15585/mmwr.mm6649a8

**Published:** 2017-12-15

Effective January 12, 2018, Announcements will be limited to public health events (e.g., World AIDS Day or Great American Smokeout) that are topically related to Full Reports. Both the Announcement and Full Report will appear on the cover of the issue. Information about other national health observances is available at https://healthfinder.gov/NHO/default.aspx.

